# Therapeutic effects of baicalein on rotenone-induced Parkinson’s disease through protecting mitochondrial function and biogenesis

**DOI:** 10.1038/s41598-017-07442-y

**Published:** 2017-08-30

**Authors:** Xue Zhang, Lida Du, Wen Zhang, Yulin Yang, Qimeng Zhou, Guanhua Du

**Affiliations:** 1 Beijing Key Laboratory of Drug Target Identification and Drug Screening, Institute of Materia Medica, Chinese Academy of Medical Science and Peking Union Medical College, 1 Xian Nong Tan Street, Beijing, 100050 China; 2 School of Biomedical Science, The Chinese University of Hong Kong, Hong Kong, S.A.R. China; 3 Guangdong Pharmaceutical University, Guangzhou, 510006 China

## Abstract

Mitochondrial dysfunction has been implicated in the pathogenesis of Parkinson’s disease (PD) for several decades, and disturbed mitochondrial biogenesis (mitobiogenesis) was recently found to be a common phenomenon in PD. Baicalein, a major bioactive flavone of *Scutellaria baicalensis Georgi*, exerted neuroprotective effects in several experimental PD models. However, the effects of baicalein in rotenone-induced PD rats and the possible mechanisms remain poorly understood. In this study, we evaluated the therapeutic effects of baicalein and explored its mechanism of action in rotenone-induced PD models. The results indicated that behavioural impairments and the depletion of dopaminergic neurons induced by rotenone were attenuated by baicalein. Furthermore, in rotenone-induced parkinsonian rats, baicalein treatment effectively restored mitochondrial function and improved mitobiogenesis, as determined by measuring the mitochondrial density and key regulators involved in mitobiogenesis. Additionally, we confirmed that baicalein enhanced mitobiogenesis through the cAMP-responsive element binding protein (CREB) and glycogen synthase kinase-3β (GSK-3β) pathways in rotenone-treated SH-SY5Y cells. Moreover, we demonstrated that the cytoprotective effects of baicalein could be attenuated by the mitobiogenesis inhibitor chloramphenicol as well as CREB siRNA transfection. Overall, our results suggested that baicalein partially enhanced mitobiogenesis to restore mitochondrial function, thus exerting therapeutic effects in rotenone-induced PD models.

## Introduction

Parkinson’s disease (PD), the second most common neurodegenerative disease, is characterized by the selective and progressive degeneration of dopaminergic neurons in the substantia nigra pars compacta, which leads to disabling motor abnormalities such as tremor at rest, rigidity, bradykinesia, and postural instability^1, 2^. Both environmental and genetic factors have been implicated in PD pathogenesis, and compelling experimental evidence implicates mitochondrial dysfunction, endoplasmic reticulum stress, oxidative damage and inflammation as crucial factors in the initiation and progression of PD^3^.

In recent decades, a substantial amount of evidence has accumulated focusing on dysfunctional mitochondria in PD etiopathogenesis^4^. There is evidence to indicate that mitochondrial bioenergetic dysfunction is an important pathogenic component in PD. For example, studies have linked dysfunction of the mitochondrial electron transport chain (ETC) complex I to PD, and many of the genes whose mutations cause familial PD are involved in mitochondrial function and homeostasis^5, 6^. More recently, growing evidence has suggested that defects in mitochondrial dynamics, including fission, fusion, transport, biogenesis, and degradation through mitophagy, may be involved in PD pathogenesis^7–9^. Mitochondrial biogenesis (mitobiogenesis), including transcription of genes encoded by both mitochondrial and nuclear genomes, is a complex process by which cells increase their mitochondrial components, ultimately increasing bioenergetic capacity^10^. Although the specific roles that alterations in mitobiogenesis play in PD pathophysiology are not entirely clear, neurotoxins (such as rotenone or 1-methyl-4-phenylpyridinium (MPTP))-induced models of PD are providing clues regarding the interplay of between mitobiogenesis and PD pathogenesis.

Rotenone, a widely used pesticide, acts as a specific mitochondrial complex I inhibitor. A growing body of evidence suggests that the rotenone model offers more advantages than other experimental PD models as it can effectively mimic the behavioural and neuropathological features of the disease through the selective degeneration of dopaminergic neurons^11, 12^. In rotenone-induced PD rats, peroxisome proliferator-activated receptor gamma coactivator-1α (PGC-1α), a strong stimulator of mitobiogenesis, is markedly downregulated, as has also been observed in PD patients. In addition, the restoration of PGC-1α levels has been shown to protect against complex I inhibition, implying that rotenone models of PD could serve as a base for exploring the relationship between mitobiogenesis and PD and evaluating neuroprotective agents against mitochondrial dysfunction^13^.

**Figure 1:**
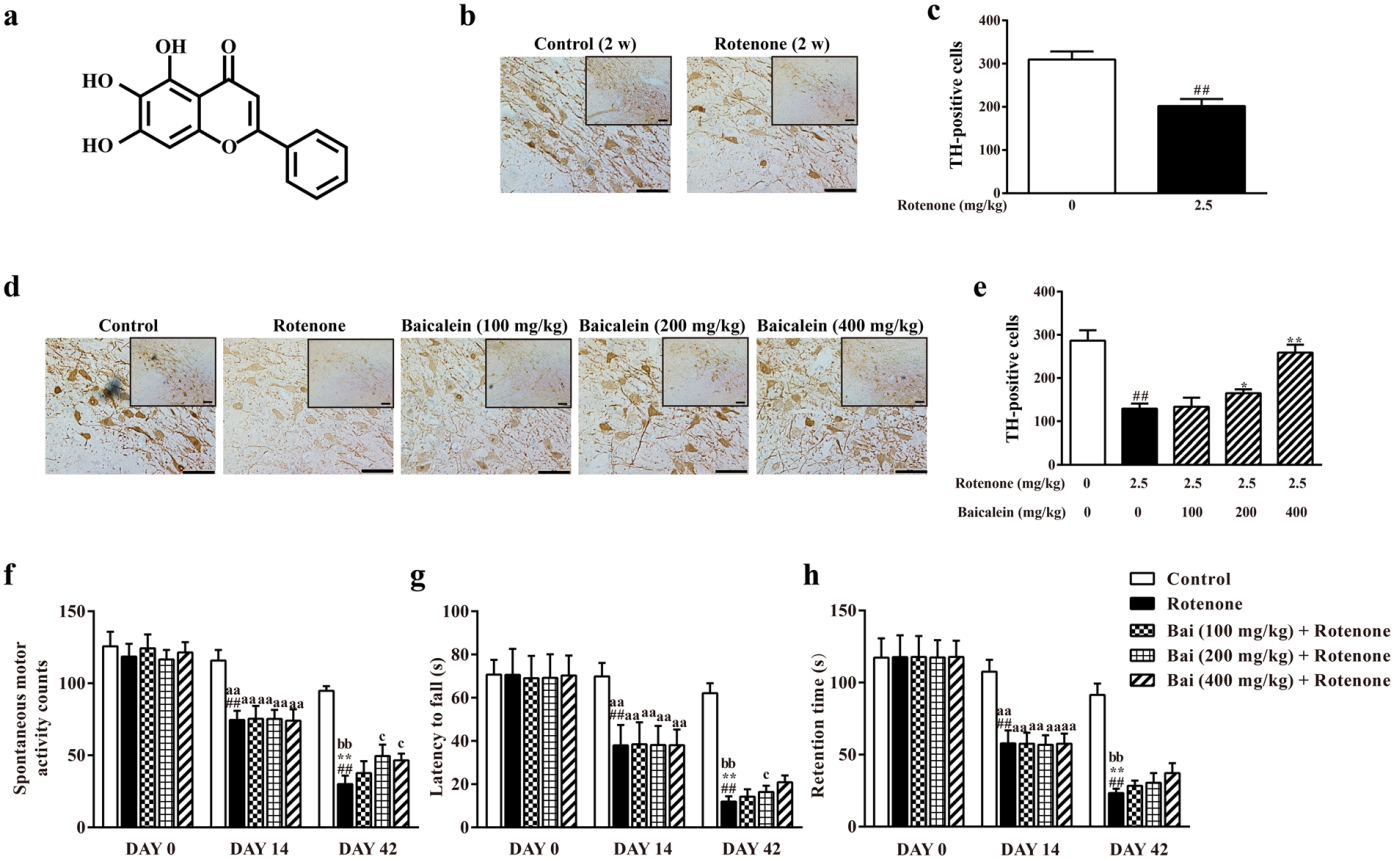
Effects of baicalein on the behavioural deficits and TH^+^ cell numbers in the SN in rotenone-induced PD rats. (**a**) Chemical structure of baicalein. The control solvent and rotenone were injected i.p. for 14 days to assess the effect of rotenone on TH^+^ cells in the SN. (**b**) Representative microphotographs of TH immunostaining and (**c**) quantification of the effect of rotenone on TH^+^ cells in the SN (scale bar: 50 μm). Values are expressed as the means ± SEMs. N = 15. Statistical analyses were performed using an unpaired sample t test. ^##^P < 0.01 compared with the control group. The effect of baicalein on the TH^+^ cell number was evaluated after administration of baicalein for 28 days along with rotenone injection for 42 days. (**d**) Representative microphotographs of TH immunostaining and (**e**) quantification of the effect of baicalein on TH^+^ cells in the SN (scale bar: 50 μm). Values are expressed as the means ± SEMs. N = 5. Statistical analyses were performed using one-way ANOVA. ^##^P < 0.01 compared with the control group, ^*^P < 0.05, ^**^P < 0.01 compared with the model group. Motor functions were assessed through a spontaneous motor activity test (**f**), rotarod test (**g**) and inclined plane test (**h**). DAY 0 and DAY 14 in the control and rotenone group: N = 15; Others: N = 10. Statistical analyses were performed using one-way ANOVA. ^##^P < 0.01 compared with DAY 0 in the rotenone group, ^**^P < 0.01 compared with DAY 14 in the rotenone group, ^aa^P < 0.01 compared with DAY 14 in the control group, ^bb^P < 0.01 compared with DAY 42 in the control group, ^c^P < 0.05 compared with DAY 42 in the rotenone group.

Baicalein (Fig. 1a), one of the major flavonoids isolated from the roots of the traditional Chinese herbal medicine Huangqin, *Scutellaria baicalensis Georgi*, has been widely used for the treatment of inflammation, hypertension, cardiovascular disease, bacterial infection and cancer. Our laboratory has focused on the role of baicalein in PD treatment for several decades, and prior data indicated that baicalein exerted a neuroprotective action against MPTP-induced damage in C57BL/6 mice, attenuated muscle tremor and increased the number of tyrosine hydroxylase (TH) neurons through its anti-oxidative action in 6-hydroxydopamine (OHDA)-lesioned rats^14–17^. Moreover, baicalein inhibited 6-OHDA-induced mitochondrial dysfunction in SH-SY5Y cells via inhibition of mitochondrial oxidation and upregulation of DJ-1 protein expression^18^. Additionally, other studies have found that baicalein attenuated astroglial activation in the MPTP-induced PD model by repressing the activation of nuclear factor-κB, extracellular-signal regulated kinases (ERK)1/2, and jun-amino-terminal kinase (JNK) and protected neurons against 6-OHDA-induced toxicity through activation of the Kelch-like ECH-associated protein 1 (Keap1)/NF-E2-related factor 2 (NRF-2)/hemo oxygenase-1 (HO-1) and Phosphoinositide 3-kinase (PI3K)/AKT signalling pathways^19, 20^. Simultaneously, experimental studies have shown that baicalein inhibits the formation of α-synuclein oligomers, and prevents the progression of α-synuclein accumulation *in vitro* and *in vivo* ^21–23^. All of these data suggest that baicalein might be effective in the prevention or treatment of PD. However, the mechanisms and target proteins underlying this protective effect remain largely unknown. In this study, we investigated the therapeutic effects of baicalein on rotenone-induced PD rats and explored whether the neuroprotective potentials exerted by baicalein were through intervening in mitochondrial function and mitobiogenesis.

## Results

### Effects of baicalein on the behavioural impairments and TH-positive cell numbers in the substantia nigra in rotenone-induced PD rats

To determine the extent of the rotenone injury, behavioural analysis was performed and TH-positive (TH^+^) cells in the substantia nigra (SN) were detected. As shown in Fig. 1(f,h and b,c), rotenone time-dependently induced behavioural impairments and reduced the TH^+^ cell numbers. Rats injected with rotenone for 14 consecutive days already exhibited significant behavioural impairments, as evidenced by lower spontaneous motor activity counts, latency to fall off of the rotarod rod and retention time on the inclined plane than on DAY 0 in the rotenone-treated rats (^##^P < 0.01, ^##^P < 0.01, ^##^P < 0.01, respectively) and DAY 14 in the control (^aa^P < 0.01, ^aa^P < 0.01, ^aa^P < 0.01, respectively), and showed a marked loss in TH^+^ neurons with 65.19% surviving TH^+^ neurons in the SN compared with that in normal controls (Fig. 1b,c). Injection of rotenone for 42 days further aggravated behavioural deficits and reduced the TH^+^ cells, with only 45.08% surviving TH^+^ neurons compared with that in normal controls (Fig. 1d,e).

However, oral administration of baicalein increased the spontaneous motor activity (200 or 400 mg/kg baicalein) and extended the latency on the rod (200 mg/kg baicalein). Additionally, baicalein dose-dependently increased the number of TH^+^ neurons, with 27.55% more TH^+^ neurons in rats treated with 200 mg/kg baicalein and 100.30% more TH^+^ neurons in those treated with 400 mg/kg baicalein compared with that observed in the PD model rats not treated with baicalein (DAY 42) (Fig. 1d,e). All of the above results suggested that baicalein partially ameliorated the motor dysfunction and increased the number of TH^+^ cells in the SN in rotenone-induced PD rats.

### Effects of baicalein on neuronal apoptosis in the SN of rotenone-induced PD rats

**Figure 2:**
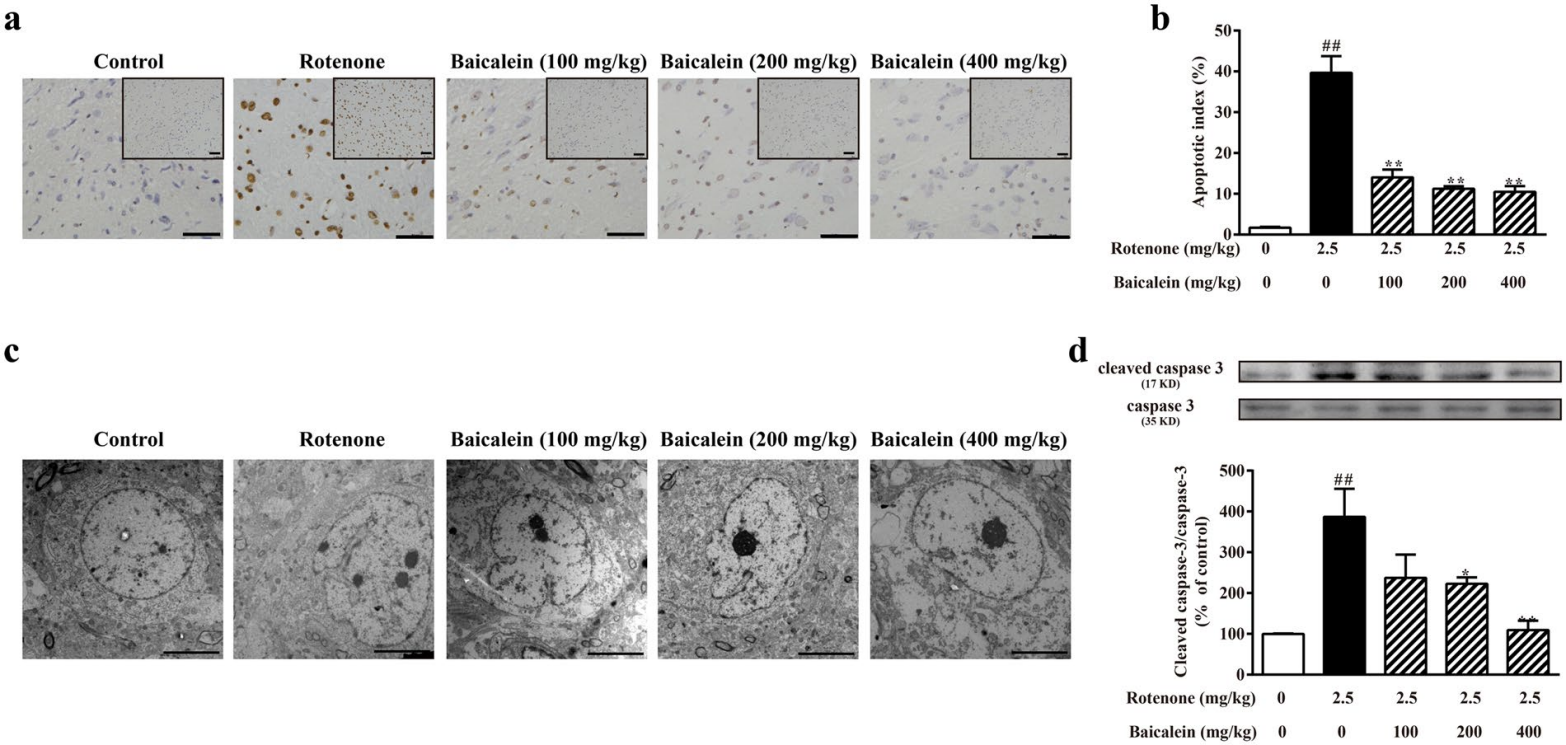
Effects of baicalein on neuronal apoptosis in the SN of rotenone-induced PD rats. (**a**) Representative microphotographs of TUNEL staining in the SN (scale bar: 50 μm). (**b**) Quantification of the effect of baicalein on TUNEL-positive cells in the SN in rotenone-induced PD rats. (**c**) The ultrastructure of neurons in the SN in rotenone-induced parkinsonian rats was observed by transmission electron microscopy (scale bar: 5 μm). (**d**) The level of cleaved caspase-3 was detected by western blotting. Values are expressed as the means ± SEMs. N = 5. Statistical analyses were performed using one-way ANOVA. ^##^P < 0.01 compared with the control group, ^*^P < 0.05, ^**^P < 0.01 compared with the model group.

TUNEL staining and caspase-3 detection were employed to evaluate neuronal apoptosis in the SN after rotenone exposure. As shown in Fig. 2a,b and d, the number of TUNEL-positive cells and the protein level of cleaved caspase-3 was significantly higher in the SN after rotenone injection for 42 days than in the control rats (^##^P < 0.01, ^##^P < 0.01, respectively). Administration of baicalein dose-dependently reduced the number of TUNEL-positive cells and the level of cleaved caspase-3.

The ultrastructural features of neurons in the SN were observed by transmission electron microscopy (Fig. 2c). After 42 days of rotenone treatment, neurons in the SN presented with obvious apoptotic features, exhibiting high condensation and margination of chromatin wrapped in a wavy-like and incomplete nuclear membrane. However, the ultrastructural features of apoptosis were ameliorated in the high dose of baicalein group, with cells showing an intact nuclear membrane, mild margination and heterochromatic chromatin. These results suggested that baicalein protected neurons in the SN against rotenone-induced apoptosis.

### Effects of baicalein on the functional alteration of mitochondria in rotenone-induced PD rats

**Figure 3:**
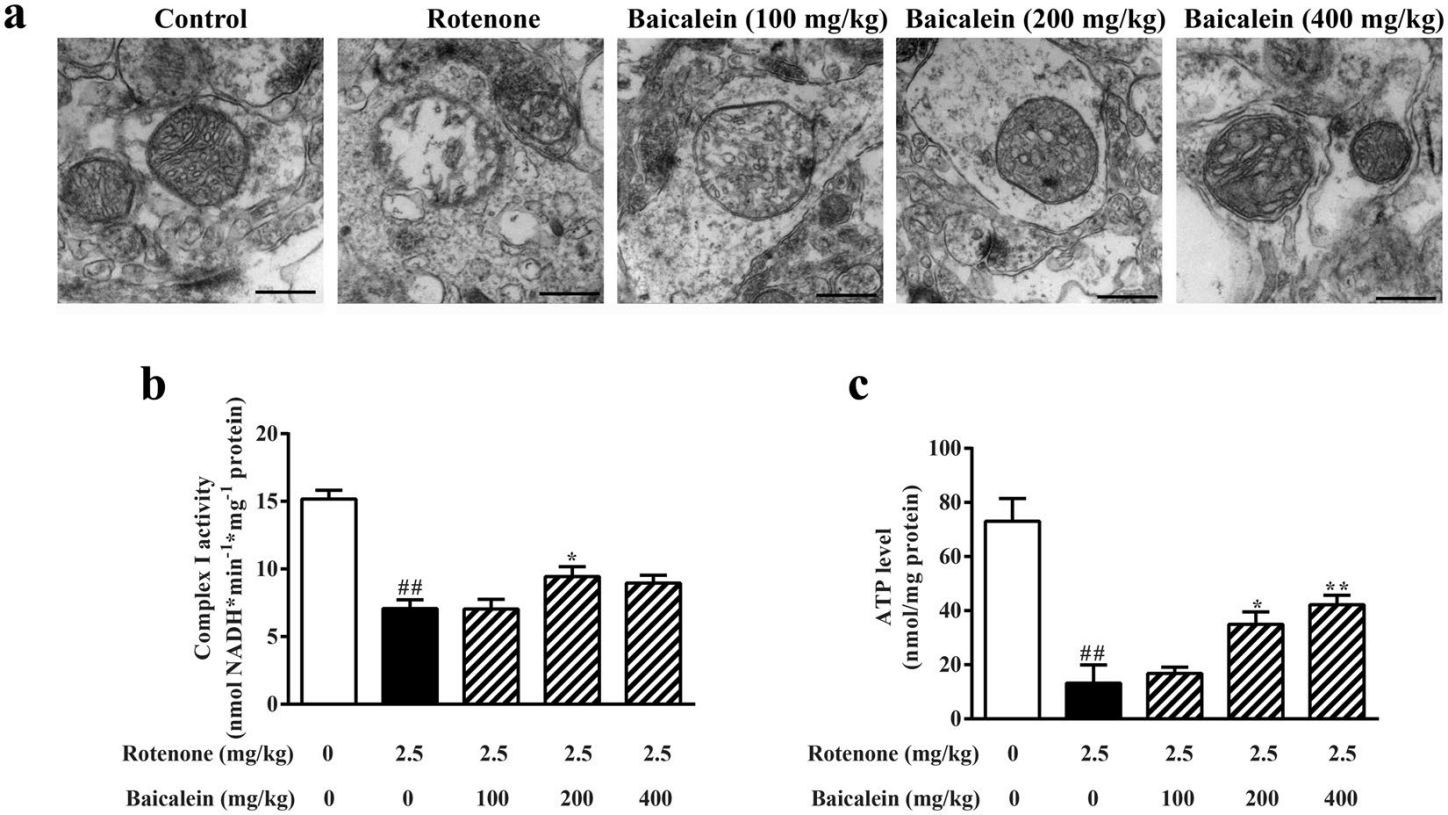
Effect of baicalein on mitochondrial ultrastructure and function after rotenone injection for 42 days in rats. (**a**) Representative photomicrographs of mitochondria in the SN were obtained by transmission electron microscopy (scale bar: 500 nm). (**b**) The activity of mitochondrial complex I in the ventral midbrain in rotenone-induced PD rats was assessed using assay kits. (**c**) ATP levels in the ventral midbrain were detected using an ATP assay kit. Values are expressed as the means ± SEMs. N = 5. Statistical analyses were performed using one-way ANOVA. ^##^P < 0.01 compared with the control group, ^*^P < 0.05, ^**^P < 0.01 compared with the model group.

As shown in Fig. 3a, the ultrastructure of mitochondria in the SN was intact in the control, with a relatively dark, uniform matrix filled with densely packed, regularly distributed cristae, whereas obvious damage had occurred in the mitochondria following rotenone exposure, which was evidenced by disrupted cristae, sometimes entirely lacking, and a loss of matrix density; some of these abnormal mitochondria appeared largely swollen and contained electron-dense inclusion bodies. Baicalein treatment restored the morphological alterations of mitochondria in the SN linked to rotenone exposure.

Mitochondrial bioenergetics in the ventral midbrain was assessed after 42 days of rotenone injection to evaluate the role of baicalein in maintaining mitochondrial respiration. Rotenone obviously inhibited the activity of complex I which was consistent with previous findings, and 200 mg/kg baicalein enhanced the activity of NADH dehydrogenase (Fig. 3b; 46.62% and 62.18% compared with that in the control group in the model and 200 mg/kg group, respectively). In addition, the ATP levels in the ventral midbrain were substantially downregulated by rotenone, whereas 400 mg/kg baicalein increased the ATP level compared with that in the model group (Fig. 3c). In this rotenone-induced PD model, as shown in Table 1, complex I-sustained mitochondrial state 3 respiration was decreased, whereas state 4 respiration remained unchanged in rotenone-treated rats. Along with a decreased state 3 respiration, mitochondria in the ventral midbrain also showed a reduced respiratory control ratio (RCR; an indicator of oxidative phosphorylation coupling and mitochondrial membrane integrity) and ADP/O values (an index of oxidative phosphorylation efficiency). However, administration of baicalein (200 or 400 mg/kg baicalein) increased state 3 and state 4 respiration, RCR and ADP/O, suggesting that baicalein ameliorated the dysfunction of mitochondrial complex I in the ventral midbrain that was damaged by rotenone.

**Table 1:** Effects of baicalein on mitochondrial respiratory function with L-glutamate plus L-malate as the substrate in rotenone-treated rats.

Group	RCR	Respiratory activity		
		ADP/O	State 3	State 4
		(nmol ADP/nmol O)	(nmol O/min/mg protein)	
Control	5.05 ± 0.41	2.16 ± 0.19	56.34 ± 1.89	11.22 ± 1.01
Rot	3.03 ± 0.27^##^	1.67 ± 0.26^##^	29.98 ± 1.47^##^	9.95 ± 0.72
Bai (100 mg/kg) + Rot	2.71 ± 0.74	1.61 ± 0.02	32.33 ± 2.18	12.67 ± 3.38
Bai (200 mg/kg) + Rot	3.37 ± 0.07^*^	2.13 ± 0.06^**^	37.70 ± 2.67^**^	11.18 ± 0.87
Bai (400 mg/kg) + Rot	3.12 ± 0.51	2.35 ± 0.20^**^	34.47 ± 5.18^**^	11.23 ± 1.48

In this table, bai and rot are short for baicalein and rotenone, respectively. Values are expressed as the means ± SEMs. N = 5. Statistical analyses were performed using one-way ANOVA. ^##^P < 0.01 compared with the control group, ^*^P < 0.05, ^**^P < 0.01 compared with the model group.

### Effects of baicalein on mitocbiogenesis in rotenone-induced PD rats

**Figure 4:**
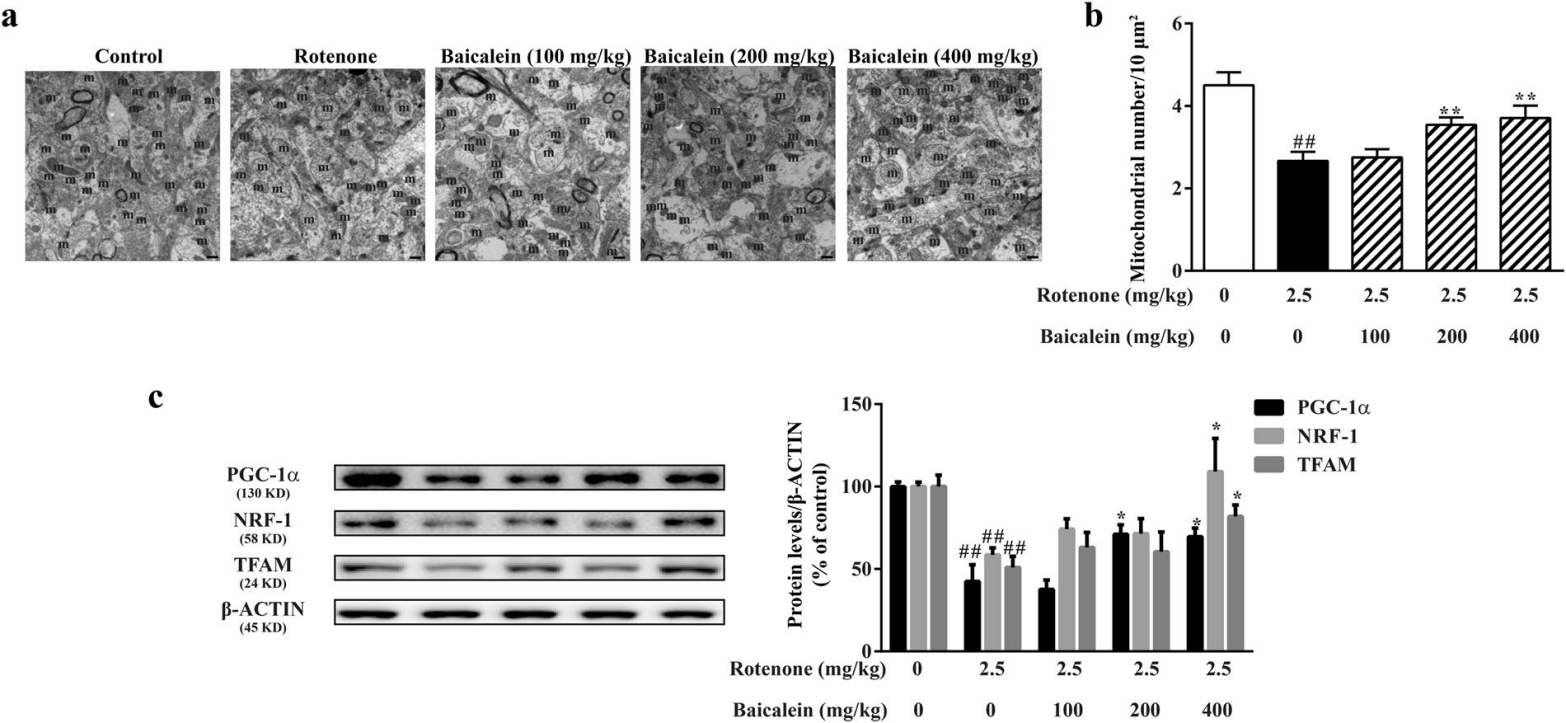
Effects of baicalein on mitobiogenesis in rotenone-induced PD rats. (**a**) Representative transmission electron photomicrographs of mitochondria in the SN. M indicates mitochondria. Scale bar: 500 nm. (**b**) Quantification of the mitochondrial number per 10 μm^2^ in the SN. For each group, N = 3 and 8 photomicrographs were counted per animal. (**c**) The expression levels of PGC-1α, NRF-1, and TFAM, key regulators of mitobiogenesis, in the ventral midbrain were examined by western blotting. All values are expressed as the means ± SEMs. N = 5. Statistical analyses were performed using one-way ANOVA. ^##^P < 0.01 compared with the control group, ^*^P < 0.05, ^**^P < 0.01 compared with the model group.

The mitochondrial number is regulated by mitobiogenesis and is a typical marker used for this process. As shown in Fig. 4a and b, the electron micrographs of the SN in the control group revealed abundant mitochondria, whereas rats in the model group had a 40.74% decrease in the mitochondrial number. Administration of baicalein (200 or 400 mg/kg) for 28 days substantially increased the mitochondrial number in the SN (78.70%, 82.41%, respectively).

PGC-1α, nuclear respiratory factor-1 (NRF-1), and mitochondrial transcriptional factor A (TFAM) are the key regulators of mitobiogenesis. As shown in Fig. 4c, the expression levels of PGC-1α, NRF-1, and TFAM were significantly lower in the model group than in the controls (^##^P < 0.01, ^##^P < 0.01, ^##^P < 0.01, respectively). However, administration of baicalein increased the protein levels of PGC-1α (200 or 400 mg/kg baicalein), NRF-1 (400 mg/kg baicalein), and TFAM (400 mg/kg baicalein) in the ventral midbrain, indicating that baicalein, especially at the 400 mg/kg dose, enhanced the mitobiogenesis that was disturbed by rotenone.

### Baicalein rescued rotenone-repressed mitobiogenesis in SH-SY5Y cells

**Figure 5:**
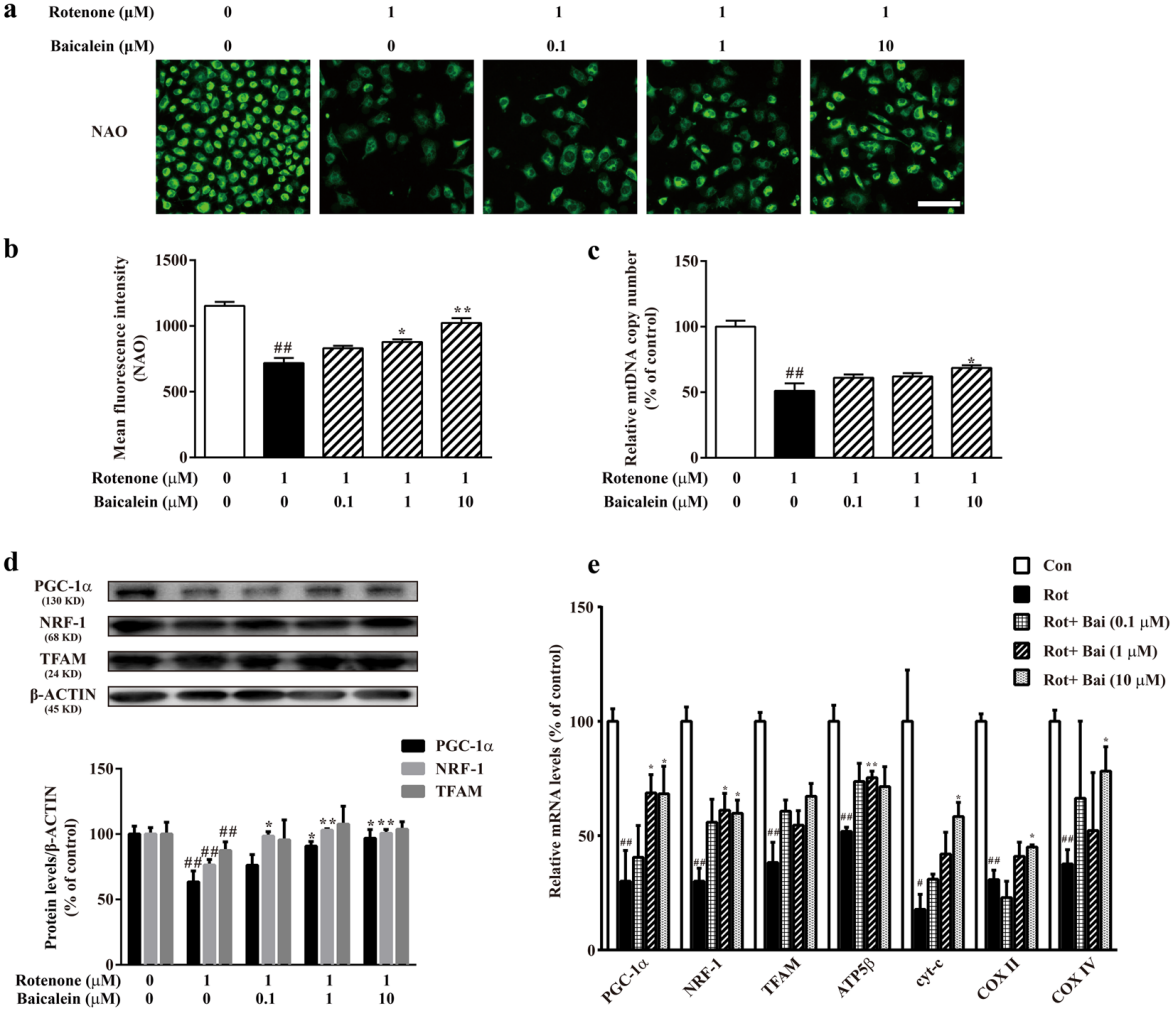
Baicalein rescued the rotenone-reduced mitochondrial abundance in SH-SY5Y cells. (**a**) Representative images of the mitochondrial mass stained by NAO (scale bar: 50 μm). (**b**) Mean fluorescence intensity was assessed based on the NAO fluorescence on the ArrayScan HCS Reader with the Morphology Explorer BioApplication. (**c**) mtDNA copy number was determined by real-time qPCR. (**d**) The expression of PGC-1α, NRF-1, and TFAM, key regulators of mitobiogenesis, was examined by western blotting. (**e**) The mRNA levels of PGC-1α and its targeted genes (NRF-1, TFAM, ATP5β, cyt-c, COX II, and COX IV) directly involved in mitobiogenesis were detected by RT-qPCR. Values are expressed as the means ± SEMs. N = 3. Statistical analyses were performed using one-way ANOVA. ^##^P < 0.01 compared with the control group, ^*^P < 0.05, ^**^P < 0.01 compared with the model group.

To determine whether baicalein indeed enhanced mitobiogenesis *in vitro* under rotenone treatment, we examined two parameters of mitobiogenesis in SH-SY5Y cells: The mitochondrial quantity was assessed through Nonyl Acridine Orange (NAO) staining and the mitochondrial DNA (mtDNA) copy number was measured by real-time PCR. As shown in Fig. 5a and b, after exposure to rotenone for 24 h, the fluorescence intensity of NAO decreased 37.68% in model cells compared with that in the controls. In addition, baicalein increased the relative intensity of NAO fluorescent dye in a concentration-dependent manner. We also observed a decrease in the mtDNA copy number in the model group compared with that in the control cells. However, baicalein (10 μM) partly rescued the decrease in mtDNA content in rotenone-treated SH-SY5Y cells (Fig. 5c).

Moreover, activators and co-activators involved in mitobiogenesis were detected. Figure 5d shows that the expression of PGC-1α, NRF-1 and TFAM significantly decreased after rotenone treatment compared with that in the controls (^##^P < 0.01, ^##^P < 0.01, ^##^P < 0.01, respectively). However, treatment with baicalein (1 μM or 10 μM) resulted in an increase in the protein expression of PGC-1α and NRF-1. These data indicated that baicalein induced expression of activators associated with mitobiogenesis in rotenone-injured SH-SY5Y cells.

As the PGC-1α protein level was influenced by baicalein, the mRNA levels of PGC-1α-targeted genes directly involved in mitobiogenesis, including NRF-1, TFAM, ATP synthase subunit-β (ATP5β), cytochrome c (cyt-c), cytochrome c oxidase (COX) II, and COX IV, were further measured. Figure 5e shows that the mRNA levels of NRF-1, TFAM, ATP5β, cyt-c, COX II, and COX IV were significantly decreased after rotenone treatment (^##^P < 0.01, ^##^P < 0.01, ^##^P < 0.01, ^#^P < 0.05, ^##^P < 0.01, ^##^P < 0.01, respectively). However, treatment with baicalein increased the transcription levels of PGC-1α, NRF-1, ATP5β, cyt-c, COX II, and COX IV, compared with those in the model group. All of the above data indicated that baicalein attenuated the rotenone-repressed mitobiogenesis in SH-SY5Y cells.

### Baicalein increased PGC-1α expression by regulating the phosphorylation of CREB and GSK-3β

**Figure 6:**
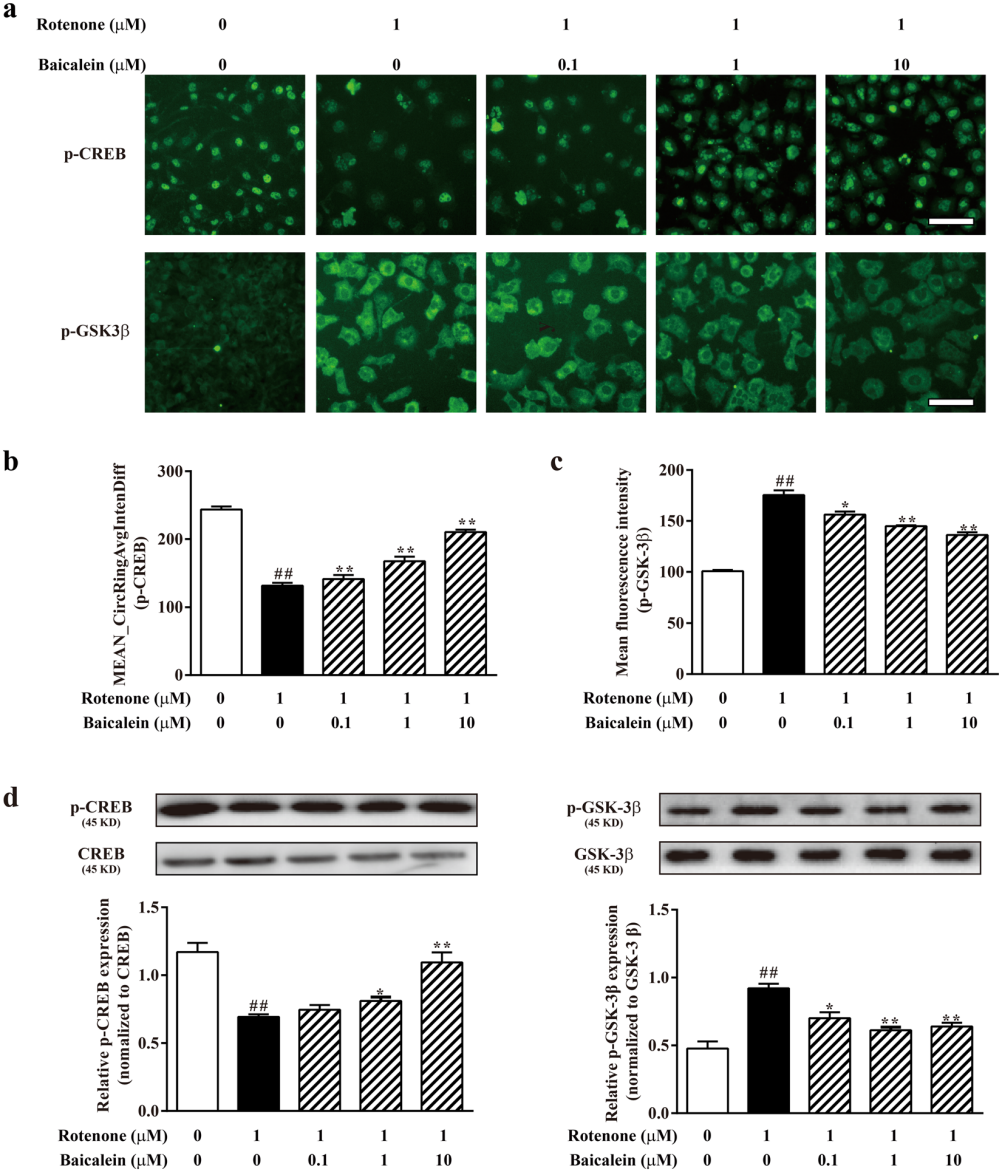
Baicalein increased PGC-1α expression by regulating phosphorylation of CREB and GSK-3β. (**a**) Representative images of p-CREB and p-GSK-3β were acquired on the ArrayScan HCS Reader (scale bar: 50 μm). (**b**) Values of the Mean CircRingAvgIntenDiff described the translocation capacity of cytosolic p-CREB to the nucleus. (**c**) The mean fluorescence intensity illustrated the levels of p-GSK-3β in the cytoplasm. (**d**) The expression of p-CREB, CREB, GSK-3β and p-GSK-3β were examined by western blotting. Values are expressed as as the means ± SEMs. N = 3. Statistical analyses were performed using one-way ANOVA. ^##^P < 0.01 compared with the control group, ^*^P < 0.05, ^**^P < 0.01 compared with the model group.

Due to the effects of baicalein on PGC-1α expression, we determined whether factors that regulate PGC-1α were regulated by baicalein. We therefore examined regulators that influence PGC-1α abundance or activity, including cAMP-responsive element binding protein (CREB), glycogen synthase kinase-3β (GSK-3β), p38, ERK1/2, AMP-activated protein kinase (AMPK) and Sirtuin1 (SIRT1). As shown in Fig. 6a and b, rotenone significantly decreased the steady-state levels of p-CREB (^##^P < 0.01 compared with the control), whereas the level of p-CREB was increased by baicalein in a concentration-dependent manner. We next investigated whether baicalein downregulated GSK-3β *in vitro*. In SH-SY5Y cells, rotenone treatment increased the p-GSK-3β abundance, but this increase was reduced by approximately 30% in 10 μM baicalein-treated cells (Fig. 6a and c).

In addition to CREB and GSK-3β, we further investigated the possible role of baicalein on changes in p-p38, p-ERK1/2, p-AMPK and SIRT1 induced by rotenone. Rotenone resulted in a significant increase in p-p38, p-ERK1/2, p-AMPK and SIRT1 protein levels (^##^P < 0.01, ^##^P < 0.01, ^##^P < 0.01, ^##^P < 0.01, respectively), but baicalein treatment did not appear to influence these protein levels in the immunofluorescence assays (Supplementary Fig. S1).

### Mitobiogenesis inhibitor and CREB siRNA blocked the cytoprotective effects of baicalein in rotenone-treated SH-SY5Y cells

**Figure 7:**
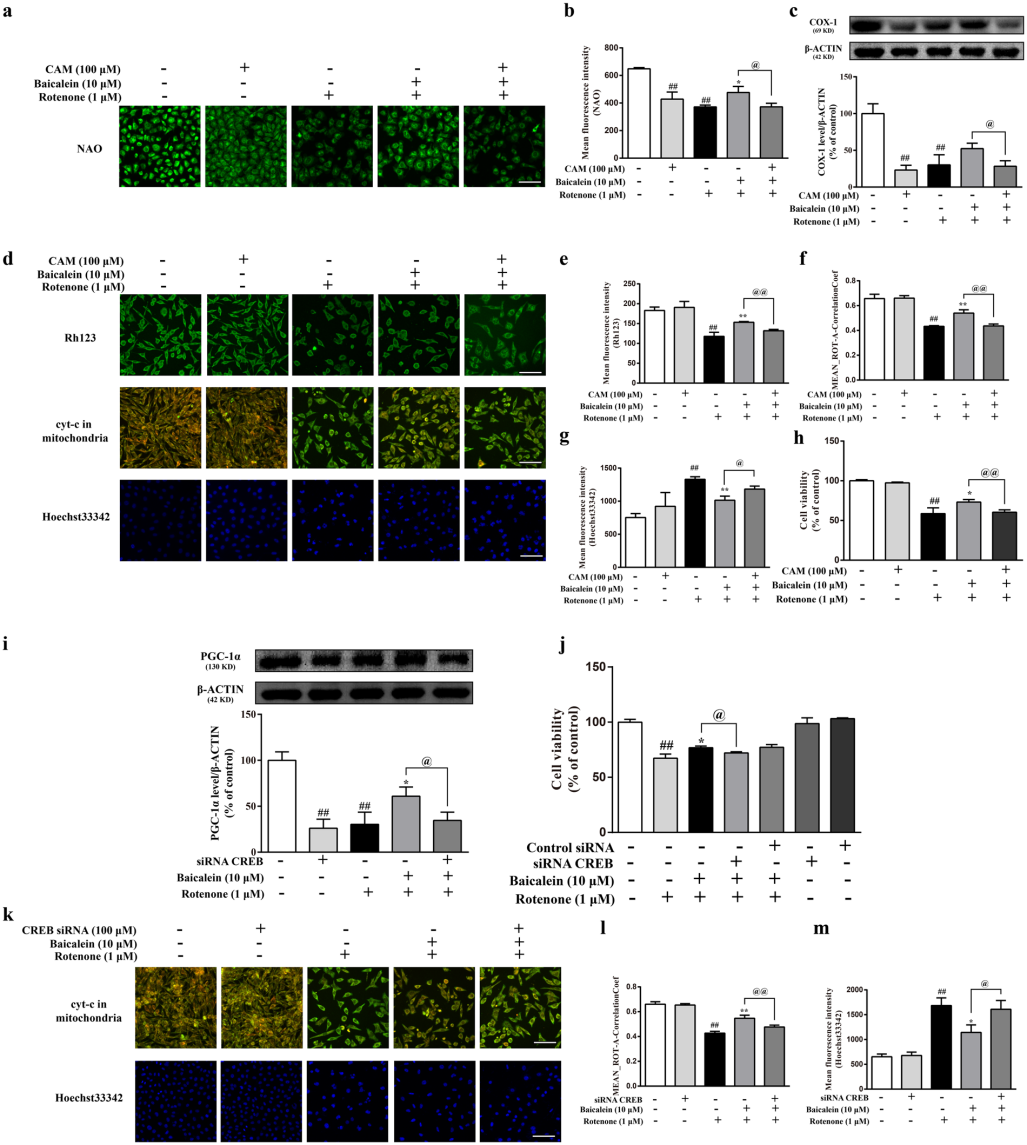
Mitobiogenesis inhibitor and CREB siRNA block the cytoprotective effects of baicalein in rotenone-treated SH-SY5Y cells. (**a**) Representative images of the mitochondrial mass stained by NAO (scale bar: 50 μm). (**b**) The mean fluorescence intensity was assessed based on the NAO fluorescence on the ArrayScan HCS Reader with the Morphology Explorer BioApplication. (**c**) The expression of COX-1 was examined by western blotting. (**d**) Representative images of MMP, cyt-c and nuclei were acquired on the ArrayScan HCS Reader. Scale bar: 50 μm. (Methods in Supplementary Information). Mean fluorescence intensity was assessed based on the Rh123 (**e**) and Hoechst33342 (**g**) fluorescence. (**f**,**l**) Values of MEAN_ROT-A-CorrelationCoef describe the colocalization of cyt-c and mitochondria. (**h**,**j**) Cell viability was detected using a CCK-8 assay. (**i**) The expression of PGC-1α was examined by western blotting. (**k**) Representative images of cyt-c and nuclei were acquired on the ArrayScan HCS Reader (scale bar: 50 μm). Values are expressed as the means ± SEMs. N = 3. Statistical analyses were performed using one-way ANOVA. ^##^P < 0.01 compared with the control group, ^*^P < 0.05, ^**^P < 0.01 compared with the rotenone group, ^@^P < 0.05, ^@@^P < 0.01 compared with the baicalein plus rotenone group.

Chloramphenicol (CAM), an inhibitor of mitochondrial biogenesis in mammalian cells, reversed the increase in COX-1 expression (a mitochondrial structural protein) and mitochondrial quality (detected by NAO staining) induced by baicalein (Fig. 7a–c; ^@^P < 0.05, ^@^P < 0.05, respectively). In terms of mitochondrial function, the inhibition of mitobiogenesis attenuated the protective effects of baicalein against the rotenone-induced mitochondrial membrane potential (MMP) decrease and cyt-c release (Fig. 7d–f; ^@@^P < 0.01, ^@@^P < 0.01, respectively). In addition to cyt-c, CAM treatment blocked the anti-apoptotic effects of baicalein, which was indicated by Hoechst33342 staining (Fig. 7d–g; ^@^P < 0.05). Additionally, the cytoprotective effects of baicalein were also suppressed by CAM pretreatment (Fig. 7h; ^@@^P < 0.01).

To better define the role of CREB associated with baicalein, we tested the effect of CREB siRNA transfection on the baicalein-induced protection against rotenone treatment in SH-SY5Y cells. We found that CREB siRNA transfection dramatically decreased PGC-1α protein expression and reversed the increases of PGC-1α expression that were induced by baicalein in rotenone-treated SH-SY5Y cells (Fig. 7i; ^##^P < 0.01, ^@^P < 0.05, respectively). Notably, silencing of the CREB protein clearly abrogated the anti-apoptotic effects of baicalein against rotenone-induced cyt-c release (Fig. 7k,i; ^@@^P < 0.01) and nuclear damage (Fig. 7k,m; ^@^P < 0.05). Additionally, in a cell viability test, CREB siRNA transfection significantly blocked the cytoprotective effects of baicalein; however, both CREB siRNA and control RNA transfection alone did not affect SH-SY5Y cell viability (Fig. 7m).

Together, these data suggested that the cytoprotective effect of baicalein was dependent on CREB-mediated mitobiogenesis.

## Discussion

In the present study, we report a therapeutic effect of baicalein on rotenone-induced PD rats and a novel role of baicalein in restoring mitochondrial function, especially mitobiogenesis. In rotenone-treated SH-SY5Y cells, baicalein triggered an increase in mitobiogenesis which was correlated with the upregulation of the key controller of mitobiogenesis, PGC-1α, through the activation of CREB and inactivation of GSK-3β. Collectively, this study provides evidence that baicalein exerts therapeutic effects partially through the activation of the CREB/GSK-3β/PGC-1α pathway to enhance mitobiogenesis and subsequently improve mitochondrial function.

As PD is both a chronic and progressive disease with its symptoms growing worse over time, the effectiveness of the long-term administration of baicalein was studied in a developing rotenone model of PD that faithfully recapitulated the progressive pathological and phenotypic features of PD within 6 weeks of its treatment. In this study, injection of rotenone for only 14 days induced parkinsonian-like symptoms in rats, including behavioural impairment and dopaminergic neuron damage (Fig. 1). Therefore, DAY 15 (when PD symptoms were induced) to DAY 42 (we monitored the general performance of the rotenone-injected rats every day, finding an improvement in the baicalein-treated groups at this timepoint) was used as the period for the therapeutic administration of baicalein. After administration of baicalein for 28 days, the rotenone-induced locomotor hypoactivity and impairments were partially attenuated. There was no significance between the rats treated with the low dose of baicalein and the model rats (DAY 42) in all three behavioural tests or between the rats treated with any dose of baicalein and the model rats (DAY 42) in the inclined plate test, which may be due to the slight efficacy of baicalein and the limited sample size. However, baicalein showed a dose-dependent trend in improvement of the motor impairments induced by rotenone. Interestingly, however, 200 mg/kg baicalein was more effective than the high dose in reversing the effects on the spontaneous motor activity test which may be related to the individual differences in the animals. Therefore, the effects of baicalein on the spontaneous motor activity and rotarod test, and the tendency of baicalein to affect activity on the inclined plane test implied that baicalein partially ameliorated the motor dysfunction in rotenone-induced parkinsonian rats. Rotenone is known to produce these PD-like behavioural features mainly by destroying the dopaminergic neurons via apoptosis^24^. In context, our results showed a significant decline in TH (dopamine biosynthetic enzyme) immunoreactivity and obvious neuronal apoptosis in the SN in rotenone-treated rats; in addition, the reduction in TH^+^ cell numbers and neuronal apoptosis were attenuated by baicalein (200 or 400 mg/kg, ig.).

Mitochondria are involved in cell survival and play a central role in apoptosis through the control of cellular energy metabolism, the generation of ROS, and the release of apoptotic factors. Mitochondrial dysfunction is an early event in almost all neurodegenerative diseases, including PD. Rotenone inhibits the activity of NADH-ubiquinone reductase, leading to the destruction of mitochondrial function and even mitochondrial structural damage, such as the disruption and dissolution of mitochondrial cristae^11, 24, 25^. Our data confirmed the defective mitochondrial function induced by rotenone mentioned above with suppressed NADH dehydrogenase activity, restrained mitochondrial respiration initiated by NADH, decreased ATP levels, and abnormal morphological mitochondria in rotenone-treated rats (Fig. 3 and Table 1), and reduced intracellular ATP, collapsed mitochondrial membrane potential and increased cyt-c release in rotenone-treated SH-SY5Y cells (Supplementary Fig. S2). In our previous studies, we have reported protective effects of baicalein against rotenone-induced toxicity in isolated rat brain mitochondria^26^. Additionally, baicalein prevented 6-OHDA-induced mitochondrial dysfunction via inhibition of mitochondrial oxidation in SH-SY5Y cells^18^. In the present study, we further explored the role of baicalein on mitochondria, finding that baicalein attenuated the rotenone-induced ETC defects, bioenergetic dysfunction, and structural impairment *in vivo* and *in vitro*, which indicated that baicalein contributed to the recovery of mitochondrial function. Additionally, in terms of complex I activity, 200 mg/kg baicalein was more effective than 400 mg/kg, whereas 400 mg/kg baicalein had a greater effect than 200 mg/kg when considering the ATP levels and mitochondrial respiration. These data indicated that baicalein might affect other aspects of mitochondria besides complex I, such as complex III/IV (which is involved in the mitochondrial respiration initiated by NADH) and mitochondrial structural protein (discussed as follows), to play a comprehensive role in mitochondrial function. Furthermore, our results implied that the effect of baicalein on mitochondrial complex I may not be direct but rather secondary.

Mitochondrial dysfunction is closely interconnected to the dysregulation of mitochondrial dynamics, and is therefore of particular importance in neurons, which have a unique bioenergetic profile due to their energetic dependence on mitochondria and specialized, compartmentalized energetic needs^27^. The mitochondrial dynamic “lifecycle” begins with the coordinated synthesis of nuclear DNA and mitochondrial DNA-encoded proteins, together with membrane biosynthesis and the proper targeting and folding of respiratory chain subunits, grouped under the term “mitobiogenesis”^28^. Normally, mitochondrial biogenesis and function are dynamically regulated to adapt to the energetic and metabolic demands in response to physiological signals. In many neurodegenerative diseases, brain mitochondrial function is impaired, and mitobiogenesis is increased to compensate for the functional decline. Neurodegeneration will occur in the case of insufficient mitobiogenesis to balance the functional declines^29, 30^. Reduced mitochondrial content has been reported to be a common phenomenon in patients with sporadic PD as well as familial models of PD, along with neuronal overexpression of the A53T mutation of α-synuclein and neuronal expression of the mutant leucine rich repeat kinase 2^31–33^.

In our study, we demonstrated that baicalein ameliorated rotenone-induced mitobiogenesis repression. First, the mitochondrial density in the brain, which was reduced in rotenone-treated rats, was increased after baicalein administration. Second, the amount of mtDNA and the mitochondrial content were decreased in rotenone-treated SH-SY5Y cells, whereas baicalein effectively alleviated these reductions. Third, we investigated several transcription factors and co-factors that are involved in the activation and regulation of mitobiogenesis, including PGC-1α, NRF-1 and TFAM. Our data showed an increased abundance of these factors after baicalein treatment in rotenone *in vivo* and *in vitro* models. In rotenone-induced parkinsonian rats, 200 and 400 mg/kg baicalein increased the PGC-1α protein level, whereas only 400 mg/kg baicalein showed a significant influence on NRF-1 and TFAM levels. These results indicated that baicalein had a much stronger influence on PGC-1α than NRF-1 and TFAM. In addition, PGC-1α is not the only regulator that functions upstream of NRF-1 and TFAM. Data from the rotenone-treated SH-SY5Y cells confirmed this conclusion. Moreover, ATP5β, cyt-c, COX II and COX IV, genes targeted by PGC-1α, are involved in mitobiogenesis directly. Our study found that the reduction in the mRNA levels of these genes was significantly attenuated by baicalein. All in all, baicalein upregulated expression of the key regulators of mitobiogenesis (PGC-1α, NRF-1 and TFAM) and PGC-1α-targeted genes that are involved in mitobiogenesis to enhance mitobiogenesis, as shown by the increased mitochondrial density and content in the rotenone models.

As discovered in this study, baicalein exerted an influence on PGC-1α, evidenced by its change in protein expression and mRNA level and the transcription levels of its targeted genes, to regulate mitobiogenesis. We thus conducted further experiments to illustrate the mechanism of the effect of baicalein on PGC-1α.

There are several major signalling pathways that function upstream to regulate the activation and bioavailability of PGC-1α. AMPK functions to upregulate PGC-1α activity by sensing changes in the AMP/ATP ratio^34^. SIRT1 deacetylates PGC-1α to promote its nuclear translocation^35^. Alternatively, SIRT1 can activate AMPK via deacetylation and the AMPK kinase LKB1, further increasing phosphorylation of PGC-1α^36, 37^. The MAPK family has multiple effects on mitobiogenesis. Exercise-induced activation of p38γ in mice serves to increase PGC-1α promoter activity, whereas overexpression of dominant-negative p38γ attenuates mitobiogenesis^38, 39^. The ERK1/2 signalling pathway regulates PGC-1α activity in a cell-type dependent manner. In hypoxic hippocampal neurons, acetyl-L-carnitine upregulates PGC-1α and NRF-1 levels through ERK1/2-dependent mechanisms^40^. However, in other studies, inhibition of ERK1/2 signalling enhanced PGC-1α expression, thus promoting mitobiogenesis in amyloid β-injected rats^41^. CREB is another potent inducer of the regulation of PGC-1α expression. Activation of the CREB pathway results in the phosphorylation of CREB (p-CREB), and p-CREB binds the CREB-response element (CRE), located proximal to the PGC-1α promoter, and activates PGC-1α transcription^42, 43^. Additionally, GSK-3β plays a role in regulating PGC-1α protein stability, negatively regulating PGC-1α by promoting its proteasomal degradation^44, 45^.

Our studies found that baicalein produced a dose-dependent enhancement of the phosphorylation of CREB in SH-SY5Y neuronal cells whereas p-CREB levels were downregulated upon rotenone treatment. The results from our immunofluorescence and western blot analyses were consistent with the previous report, showing lower abundance of PGC-1α and a higher level of phosphorylated GSK-3β in rotenone-treated SH-SY5Y cells, whereas baicalein downregulated the p-GSK-3β expression. Thus, our data implied that baicalein increased the level of PGC-1α by upregulating the p-CREB and suppressing the phosphorylation of GSK-3β. Furthermore, we demonstrated that the cytoprotective effects of baicalein could be attenuated by the mitobiogenesis inhibitor CAM as well as CREB siRNA transfection. CAM treatment blocked the protective effects of baicalein against rotenone-induced mitochondrial dysfunction and apoptosis, indicating that the cytoprotective role of baicalein is dependent on mitobiogenesis. Moreover, silencing CREB clearly abrogated the increase in PGC-1α expression (indicating enhanced mitobiogenesis) and the anti-apoptotic effects of baicalein against rotenone, implying that the cytoprotective role of baicalein is associated with CREB-mediated mitobiogenesis.

In addition, the present study showed that 10 μM rotenone could induce overexpression of SIRT1 (Supplementary Fig. S1). This finding was consistent with a study that showed that higher expression of SIRT1 caused significant mitochondrial abnormalities (i.e., abnormal morphology of mitochondria and abnormal gene expression related to mitochondria), whereas lower expression of SIRT1 showed the opposite effect^46^. Low expression of SIRT1 may induce mitobiogenesis via deacetylation of PGC-1α, but higher SIRT1 overexpression may induce hyperdeacetylation of histone proteins and repress PGC-1α transcription, which will subsequently decrease mitochondrial mass and function. Additionally, we confirmed that 10 μM rotenone could induce a higher level of phosphorylated AMPK (Supplementary Fig. S1). These alterations in p-AMPK and PGC-1α were different from results obtained in other studies, in which activation of AMPK functioned to upregulate PGC-1α activity^47, 48^. These differences might be due to the different selected dosages and treatment durations, which may activate or inactivate signal pathways by regulating mitobiogenesis. The preliminary immunofluorescence assays showed that baicalein did not influence the changes in p-AMPK, SIRT1, p-p38 and p-ERK1/2 induced by rotenone (Supplementary Fig. S1). Nonetheless, the effect of baicalein on AMPK, SIRT1, p38 and ERK1/2 requires further study.

In the present study, we evaluated the therapeutic effects of baicalein against rotenone-induced parkinsonian-like symptoms in rats and explored the neuroprotective potential exerted by baicalein through its intervention in mitochondrial function and mediation of mitochondrial biogenesis. We verified the importance of mitobiogenesis in PD pathology and considered PGC-1α as a possible target for alleviating neuronal damage in PD models. Overall, our findings indicated that baicalein exerts therapeutic effects partially through the activation of the CREB/GSK-3β/PGC-1α pathway to enhance mitobiogenesis and subsequently improve mitochondrial function.

## Materials and Methods

### Chemicals and antibodies

Baicalein was prepared by the Institute of Materia Medica, Chinese Academy of Medical Sciences and Peking Union Medical College. The purity of baicalein was more than 98%, tested using high-performance liquid chromatography. Rotenone was purchased from Sigma-Aldrich (St. Louis, MO, USA). All antibodies used in this study are listed in Supplementary Table S1.

### Animals and treatment

Sprague-Dawley (SD) rats (male, 240–260 g) were purchased from Beijing HFK Bioscience Co., Ltd. (license: SCXK (JING) 2014–0004). All animals were housed under standard laboratory conditions. All experiments were approved by the Ethics Review Committee for Animal Experimentation for Institute of Materia Medica, Chinese Academy of Medical Sciences & Peking Union Medical College. Animal experiments were carried out in accordance with the National Institutes of Health Guide for the Care and Use of Laboratory Animals (NIH Publications No. 8023, revised 1978).

SD rats were randomized and divided into the following groups: control group (1 ml/kg/day sunflower oil i.p. for 42 days, n = 15), rotenone group (2.5 mg/kg/day in sunflower oil i.p. for 42 days, n = 15), rotenone (as group II) and low dose of baicalein (100 mg/kg, p.o., from day 15 to day 42, n = 10) group, rotenone (as group II) and middle dose of baicalein (200 mg/kg, p.o., from day 15 to day 42, n = 10) group, rotenone (as group II) and high dose of baicalein (400 mg/kg, p.o., from day 15 to day 42, n = 10) group. Behavioural studies were performed on day 0, 14 and 42 of the study. After the behavioural assessments on day 14, five rats from the control and rotenone group were anaesthetized and perfused for the immunohistochemical evaluation of TH-immunopositive neurons in the SN to determine the extent of the rotenone-induced parkinsonian-like symptoms. After the last behavioural assessments on day 42, the animals were sacrificed, and the brains were harvested quickly for the neurochemical and immunohistochemical estimation.

### Cell culture and treatment

Human neuroblastoma SH-SY5Y cells were grown as monolayers in Dulbecco’s modified Eagle’s medium with 10% heat-inactivated foetal bovine serum. SH-SY5Y cells were treated with baicalein (0.1, 1, 10 μM), and incubated with 1 μM rotenone or vehicle control for 24 h.

### Behavioural assessments

The spontaneous motor activity was measured using a computerized locomotion detection system equipped with infrared sensors (manufactured by the Institute of Materia Medica, Chinese Academy of Medical Sciences and Peking Union Medical College). Rats were individually placed in a transparent plexiglass cylinder (40–13 cm, diameter-height) and allowed to habituate to the environment for 5 min before the test, and then the numbers of horizontal and vertical movements were recorded for 10 min. The results were expressed as 10-min cumulative counts^49^.

The rotarod test was performed using a rotarod apparatus (manufactured by the Institute of Materia Medica, Chinese Academy of Medical Sciences and Peking Union Medical College). Rats were first conditioned on a stationary rod (3 cm in diameter) for 30 s and during this time any animal that fell was placed back onto the rod. Rats were next conditioned at a constant speed of 15 rpm for a period of 120 s. Rats that failed the first conditioning were given two additional conditioning periods. Thirty minutes after the last conditioning, animals were placed on the rod and timed to determine their locomotor skill, using a constant speed of 15 rpm and a cutoff limit of 120 s, three times with an interval of 30 min between each round^50^.

The inclined plane assessment was performed by placing the rats on the coarse surface of an inclined plane at a 60° angle. The duration that each rat stayed on the inclined plane was recorded. If a rat stayed on the inclined plane for more than 3 min, the score was recorded as 180 s. This test was performed in triplicate for each animal^51^.

### Immunohistochemistry

Immunohistochemistry was performed as previously described^17^. Rats were anaesthetized and perfusion-fixed with 4% paraformaldehyde after the last behavioural assessment. Brains were removed and post-fixed in paraformaldehyde, after which they were transferred into 30% sucrose until infiltration was complete. Then, coronal sections (30 μm thickness) were cut through the SN using a microtome (Leica, Germany) and incubated with 3% H_2_O_2_ to remove endogenous peroxidase activity followed by blocking with goat serum. After the sections were incubated with the anti-TH antibody, they were treated with a secondary antibody. Finally, the sections were incubated with 3,4-diaminobenzidine (DAB). The results were analysed by counting the number of positive cells at a magnification of ×200 on a Nikon microscope. The average number of positive cells was used to represent the cell density.

### TUNEL staining

DNA fission associated with apoptosis was analysed using the TUNEL detection kit (Beyotime Institute of Biotechnology, Shanghai, China). After the rats were anaesthetized and perfused, brains were isolated, dehydrated and embedded in wax. Serial sections of 10 μm thickness were cut, and sections at the level of SN were selected for the TUNEL assay. Briefly, the brain sections were prepared for labelling reaction by re-hydration followed by nuclear stripping (with proteinase K). Then, the specimens were quenched in 3% H_2_O_2_ buffer followed by equilibration with TdT buffer. This process was followed by stop/wash and anti-digoxigenin-peroxidase steps. Colour was developed using DAB to generate a brown reaction product. The number of apoptotic neurons and their percentage out of total neurons were counted under a light microscope. For each slide, five fields were randomly selected for the apoptotic index (AI) calculation at a magnification of ×200. The AI was determined as the number of apoptotic neurons divided by the total number of neurons counted ×100%, with assays performed in a blinded manner.

### Ultrastructure detection by transmission electron microscopy

After the rats were anaesthetized and perfused, the SN was isolated and cut into approximately 1-mm cubes. The samples were fixed with a solution of 2.5% glutaraldehyde with 2% paraformaldehyde, and then exposed to 1% osmium tetroxide. After several subsequent washes, the tissue samples were dehydrated with gradient alcohol, embedded in Epon and finally polymerized. Regions of interest were identified and the ultrathin sections (40–70 nm) were spread on the grids. After the sections were contrasted with uranyl acetate and lead citrate, observations were performed using a transmission electron microscopy (H-7650, HITACHI, Tokyo, Japan).

### Assessments of mitochondrial function

Mitochondria in the brain tissue were isolated by differential centrifugation (see Supplementary Information).

For the measurement of mitochondrial complex I activity, a microplate assay kit (GENMED, shanghai, China) was used following the manufacturer’s instructions, with the activity normalized by the mitochondrial protein concentration.

Mitochondrial respiration measurements were made at 30 °C using a Clark-type oxygen electrode. The measurement was carried out in a medium containing mitochondrial respiration buffer (225 mM sucrose, 5 mM K_2_HPO_4_, 10 mM Tris-HCl, 10 mM KCl, 2 mM EDTA, 100 μg/ml BSA and pH 7.4) which was preheated to 30 °C; Mitochondria (1 mg) were added to the reaction chamber and the total volume was adjusted to 500 μl. Mitochondrial state 2 respiration was initiated with glutamate (5 μl; 2 M) plus malate (5 μl; 1 M) (mitochondrial energization through complex I). ADP (10 μl; 0.5 M) was added to initiate state 3 respiration. State 4 respiration was defined as oxygen consumption after ADP consumption. The respiration rates of state 3 and state 4 respiration were calculated by the rates of oxygen consumption (nmol O_2_/ml/min). The RCR was calculated as the ratio between state 3 over state 4. The ADP/O ratio was expressed as the ratio between the amount of ADP added and oxygen consumed during state 3 respiration.

ATP levels in the ventral midbrain lysates were measured using an ATP assay kit (Beyotime Institute of Biotechnology, Shanghai, China) and were corrected by the protein level.

### Assessments of mitobiogenesis in rotenone-treated SH-SY5Y cells

The mitochondrial mass was measured using the fluorescent dye NAO (Thermo Fisher Scientific Inc., Carlsbad, CA, USA). NAO was added to SH-SY5Y cells to achieve a final concentration of 10 μM for 10 min at 37 °C after the cells were treated. The fluorescence intensity was detected and analysed with a Cellomics ArrayScan VTI HCS Reader (Cellomics Inc., Pittsburgh, PA, USA) with the Morphology Explorer BioApplication software. The mitochondrial mass was quantified by the value of the average fluorescent intensity of NAO.

Quantification of mtDNA was carried out using real-time qPCR. Total DNA was extracted using a DNAiso Reagent Kit (TaKaRa, Liaoning, China), followed by qPCR using SYBR^®^ Fast qPCR Mix (TaKaRa, Liaoning, China). The relative mtDNA copy number was calculated as the ratio of mtDNA/nDNA. The primer sequences are listed in Supplementary Table S2.

For reverse transcription (RT)-qPCR, total RNA was extracted using the TRIzol reagent (Life Technologies, Rockville, MD, USA). RT-qPCR was performed using the PrimeScript™ RT Reagent Kit (Perfect Real Time) and SYBR^®^ Fast qPCR Mix in accordance with the manual guide. β-ACTIN mRNA was used as the internal control. The primer sequences are listed in Supplementary Table S2.

### Chloramphenicol treatment

SH-SY5Y cells were seeded with 8 × 10^3^ cells per well in 96-well plates or 2 × 10^5^ cells per well in six-well plates with growth medium. Until they achieved 40–60% confluence after incubation, cells were treated with chloramphenicol (100 μM) for 24 h followed by drug treatment. After 24 h, the COX-1 level, the cyt-c in the mitochondria, NAO, Rh123 and Hoechest33342 staining, and CCK-8 assay were tested to determine the mitochondrial quality, mitochondrial function, apoptosis and cell viability.

### CREB siRNA Transfection

SH-SY5Y cells were seeded with 8 × 10^3^ cells per well in 96-well plates or 2 × 10^5^ cells per well in six-well plates with growth medium. Until they achieved 40–60% confluence after incubation, cells were transfected with CREB siRNA or control siRNA using siRNA transfection reagent according to the manufacturer’s instructions (Santa Cruz Biotechnology, Santa Cruz, CA). After drug treatment, the expression of PGC-1α, Rh123 and Hoechest33342 staining, and CCK-8 assay were tested to determine the mitobiogenesis, mitochondrial function, apoptosis and cell viability.

### Cellular immunofluorescence staining

The possible regulators of PGC-1α, including CREB, GSK-3β, p38, ERK1/2. AMPK and SIRT1, were detected by immunofluorescence and quantified on the Cellomics ArrayScan VTI high-content analysis platform^52^. Briefly, cells were incubated with the primary antibody, followed by AlexaFluor 488-conjugated goat anti-rabbit/mouse secondary antibodies. The fluorescence images were acquired using the Cellomics ArrayScan VTI HCS Reader with the Cytoplasm to Nucleus Translocation BioApplication (p-CREB, p-p38, p-ERK1/2) and the Morphology Explorer BioApplication (p-GSK-3β, p-AMPK, SIRT1).

### Western blot analysis

The brain and SY5Y cell lysates were prepared according to the standard protocol using RIPA buffer (Cell Signaling Technology, Danvers, MA, USA). Antibodies anti-caspase 3, anti-PGC-1α, anti-NRF-1, anti-TFAM, anti-p-CREB, anti-CREB, anti-p-GSK-3β, anti-GSK-3β and anti-COX-1 were used as primary antibodies. Signals were detected with the enhanced chemiluminescence detection method (CWBIO BIOTECH, Beijing, China). β-ACTIN was used as the loading control. The phosphorylation level of the selected protein was expressed as the ratio of the phosphorylated protein to total protein.

### Statistical analysis

The results are expressed as the means ± S.E.Ms. Statistical significance among multiple experimental groups was determined by one-way ANOVA followed by the Student-Newman-Keuls test. Differences between the means of two groups were compared using an unpaired sample t test. P values < 0.05 were considered statistically significant.

### Data availability

All data generated or analysed during this study are included in this published article (and its Supplementary Information files).

## Electronic supplementary material

Supplementary Information
