# Supplementary material for: Therapeutic effects of baicalein on rotenone-induced Parkinson’s disease through protecting mitochondrial function and biogenesis
Source: Sci Rep. 2017 Aug 30;7:9968. doi: 10.1038/s41598-017-07442-y (PMC5577282; doi:10.1038/s41598-017-07442-y)
Supplement: Supplementary file 1 — Supplementary Information [file 41598_2017_7442_MOESM1_ESM.pdf]

Therapeutic effects of baicalein on rotenone-induced Parkinson's disease through protecting mitochondrial function and biogenesis

Xue Zhang[1], Lida Du[2], Wen Zhang[1], Yulin Yang[3], Qimeng Zhou[1] & Guanhua Du[1*]

[1]Beijing Key Laboratory of Drug Target Identification and Drug Screening, Institute of Materia Medica, Chinese Academy of Medical Science and Peking Union Medical College, 1 Xian Nong Tan Street, Beijing, 100050, China.

[2]School of Biomedical Science, The Chinese University of Hong Kong, Hong Kong S.A.R., China.

[3]Guangdong Pharmaceutical University, Guangzhou, 510006, China.

[*]Correspondence and requests for materials should be addressed to G.D. (email: dugh@imm.ac.cn)

Supplementary Figures

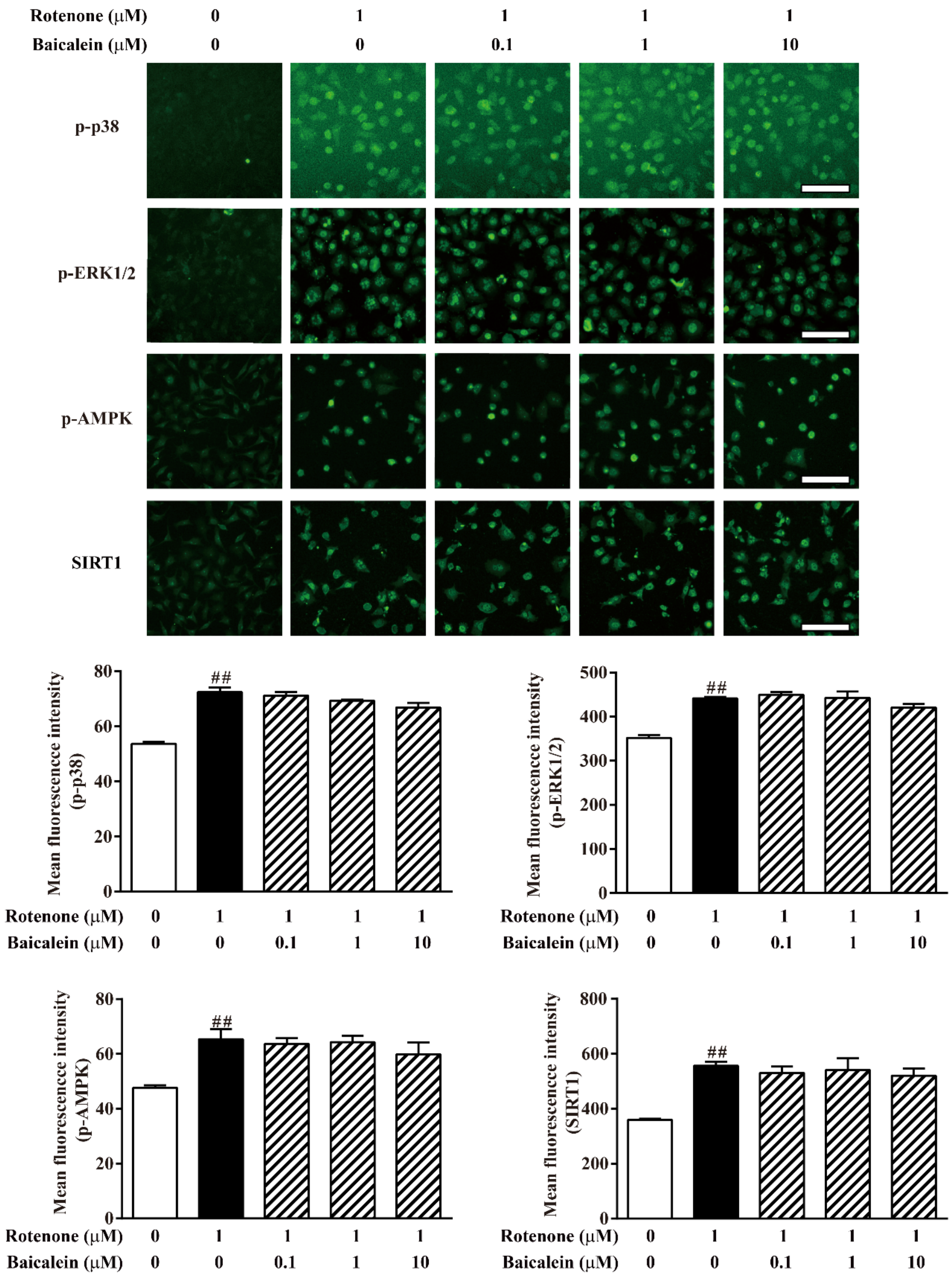

Supplementary Figure S1. Effects of baicalein on the activation of p38, ERK1/2, p-AMPK and SIRT1, possible upstream regulators of PGC-1α, in rotenone-injured SH-SY5Y cells. Representative images of p-p38, p-ERK1/2, p-AMPK and SIRT1 were acquired on the ArrayScan HCS Reader (Scale bar: 50 μm). The mean fluorescence intensity illustrated the levels of p-p38, p-ERK1/2, p-AMPK and SIRT1 in cytoplasm. Values are expressed as means ± SEM. N=3. Statistical analyses were

performed using one-way ANOVA. $^{\#\#}$ P < 0.01 compared to the control group, * P < 0.05, ** P < 0.01 compared to the model group.

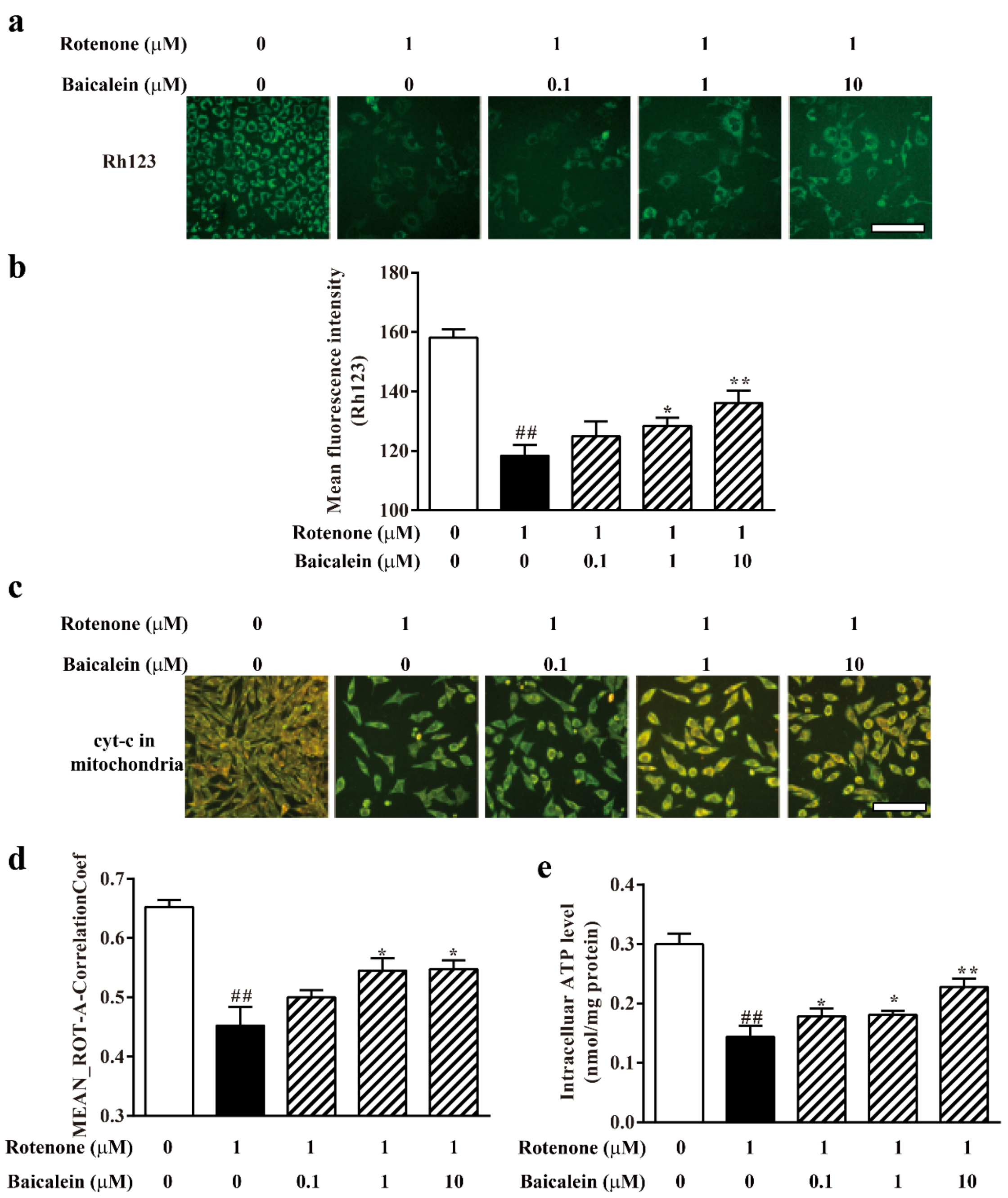

Supplementary Figure S2. Baicalein restored rotenone-damaged mitochondrial function in SH-SY5Y cells. (a) Representative images of mitochondrial membrane potential stained by Rh123 (Scale bar: 50 μm). (b) The mean fluorescence intensity was assessed based on the Rh123 fluorescence on the ArrayScan HCS Reader with the Morphology Explorer BioApplication. (c) Representative images of mitochondria and cyt-c were acquired on the ArrayScan HCS Reader using the Colocalization BioApplication. (d) Values of MEAN_ROT-A-CorrelationCoef describe the

colocalization of cyt-c and mitochondria. (e) Cellular ATP levels were detected using an ATP assay kit. Values are expressed as means ± SEM. N=3. Statistical analyses were performed using one-way ANOVA. $^{\#\#}$ $P < 0.01$ compared to the control group, * $P < 0.05$, ** $P < 0.01$ compared to the model group.

Supplementary Tables

Supplementary Table S1. Antibodies used in the study

Primary antibody	Company	Catalog#	Dilution
TH	Santa Cruz	sc-25269	1:50
cyt-c	Cell Signaling	12963	1:300
caspase 3	Cell Signaling	9662	1:1000
p-CREB (Immunofluorescence)	Cell Signaling	9198	1:800
p-CREB (Immunoblot)	Cell Signaling	9198	1:1000
CREB (Immunoblot)	Cell Signaling	9197	1:1000
p-GSK-3β (Immunofluorescence)	Cell Signaling	9323	1:100
p-GSK-3β (Immunoblot)	Cell Signaling	9323	1:1000
GSK-3β (Immunoblot)	Cell Signaling	12456	1:1000
p-p38	Cell Signaling	4511	1:3000
p-ERK1/2	Cell Signaling	4370	1:200
SIRT1	Cell Signaling	9475	1:400
β-ACTIN	Cell Signaling	3700	1:1000
AMPK	Abcam	ab23875	1:1000
PGC-1α	Abcam	ab54481	1:1000
NRF-1	Abcam	ab175932	1:2000
TFAM	Abcam	ab131607	1:2000
COX-1	Abcam	ab109065	1:2000

Alexa Fluor 488 conjugated goat anti-rabbit secondary antibody	Invitrogen	R37116	1:1000
Alexa Fluor 488 conjugated goat anti-mouse secondary antibody	Invitrogen	R37120	1:1000
Goat Anti-Rabbit IgG, HRP Conjugated	cwbiotech	CW0103S	1:1000
Goat Anti-Mouse IgG, HRP Conjugated	cwbiotech	CW0102S	1:1000

Supplementary Table S2. Primers used for real-time qPCR

Target genes	Forward primers (5’-3’)	Forward primers (5’-3’)
ND1	CACCCAAGAACAGGGTTTGT	TGGCCATGGGTATGTTGTTAA
18S nDNA	TAGAGGGACAAGTGGCGTTC	CGCTGAGCCAGTCAGTGT
PGC-1α	TCCTCACAGAGACACTAGACA	CTGGTGCCAGTAAGAGCTTCT
NRF-1	CTTACAAGGTGGGGGACAGA	GGTGACTGCGCTGTCTGATA
TFAM	CCGAGGTGGTTTTCATCTGT	TCCGCCTATAAGCATCTTG
ATP5β	GCACGGAAAATACAGCGTTT	GCCAGCTTATCAGCTTTTGC
cyt-c	GGTGATGTTGAGAAAAGGCAAG	GTTCTTATTGGCGGCTGTGT
COX II	TTCATGATCACGCCCTCATA	TAAAGGATGCGTAGGGATGG
COX IV	CCGCGCTCGTTATCATGTG	CGTTCTTTTCGTAGTCCCACTTG
β-ACTIN	AAACCCATCACCATCTTCCAG	AGGGGCCATCCACAGTCTTCT

Supplementary Methods

Preparation of mitochondria

Mitochondria in the brain tissue was isolated by differential centrifugation with

minor modifications [1]. Briefly, the freshly removed ventral midbrain tissue rapidly placed in ice-cold PBS to dislodge impurities, and homogenized in mitochondrial isolation buffer (20 mM HEPES, pH 8.0, 10 mM Tris-HCl, 1 mM EDTA, 250 mM sucrose, 1 mM PMSF, 10 g/ml leupeptin, 10 g/ml aprotinin, and 0.2 mM sodium orthovanadate) on ice. The homogenate was pelleted by centrifugation at 1000 g for 10 min at 4°C and the supernatant was continuously centrifuged at 12,000 g for 10 min at 4°C. Then pellet was washed with mitochondrial isolation buffer and spun under the same conditions to obtain the isolated mitochondria pellet, followed by resuspending in mitochondrial isolation buffer. Protein concentration of the mitochondrial suspension was determined using the BCA protein assay.

Mitochondrial membrane potential (MMP) assay

MMP was measured using the fluorescent dye Rh123 (Thermo Fisher Scientific Inc., Carlsbad, CA, USA). Rh123 was added to SH-SY5Y cells to achieve a final concentration of 10 μM for 30 min at 37°C after the cells have been treated. The intensity of fluorescence was detected and analyzed by a Cellomics ArrayScan VTI HCS Reader (Cellomics Inc., Pittsburgh, PA, USA) provided with the Morphology Explorer BioApplication software. MMP level was quantified by the value of average fluorescent intensity.

Colocalization of cytochrome c (cyt-c) and mitochondria assay

After the corresponding treatment, SH-SY5Y cells were stained with MitoTracker Orange (10 μM for 30 min at 37°C), fixed, permeabilized and stained for cyt-c using a mouse monoclonal anti-cyt-c antibody combined with Alexa Fluor 488 conjugated goat anti-mouse secondary antibody followed by Hoechst33342 nuclear staining. The intensity of fluorescence was detected and analyzed by a Cellomics ArrayScan VTI HCS Reader (Cellomics Inc., Pittsburgh, PA, USA) provided with the Colocalization BioApplication using the 549 nm excitation/620 nm emission (channel 1), 485 nm excitation/535 nm emission filters (channel 2), and 380 nm excitation/450 nm emission filters (channel 3), respectively. Briefly, images were acquired in independent channels with fixed exposure times. The entire cell was visualized by the mitochondrial stain MitoTracker Orange and we also removed the nuclear region indicated by

Hoechst33342 to give a true cytoplasmic region. Meanwhile, the individual mitochondria was stained by MitoTracker Orange and distribution of cyt-c was reflected by immunostaining. The colocalization of cyt-c with mitochondria was captured and reported by “MEAN_ROT_A_CorrelationCoef”.

1. Clark R. S. *et al.* boc-Aspartyl(OMe)-fluoromethylketone attenuates mitochondrial release of cytochrome c and delays brain tissue loss after traumatic brain injury in rats. *J Cereb Blood Flow Metab* **27,** 316-326 (2007).
